# Bioenergetic Crosstalk between Mesenchymal Stem Cells and various Ocular Cells through the intercellular trafficking of Mitochondria

**DOI:** 10.7150/thno.46332

**Published:** 2020-06-05

**Authors:** Dan Jiang, Fang-Xuan Chen, Heng Zhou, Yang-Yan Lu, Hua Tan, Si-Jian Yu, Jing Yuan, Hui Liu, Wenxiang Meng, Zi-Bing Jin

**Affiliations:** 1 Laboratory for Stem Cell and Retinal Regeneration, Institute of Stem Cell Research, Division of Ophthalmic Genetics, the Eye Hospital, Wenzhou Medical University; National Center for International Research in Regenerative Medicine and Neurogenetics, Wenzhou, 325027 China.; 2 State Key Laboratory of Molecular Developmental Biology, Institute of Genetics and Developmental Biology, Chinese Academy of Sciences, Beijing, 100101 China.; 3 Beijing Institute of Ophthalmology, Beijing Tongren Eye Center, Beijing Tongren Hospital, Capital Medical University, Beijing Ophthalmology and Visual Science Key Laboratory, Beijing, 100730 China.

**Keywords:** mitochondrial transfer, mesenchymal stem cell, corneal endothelium, photoreceptor, retinal pigment epithelium

## Abstract

**Rationale:** Mitochondrial disorders preferentially affect tissues with high energy requirements, such as the retina and corneal endothelium, in human eyes. Mesenchymal stem cell (MSC)-based treatment has been demonstrated to be beneficial for ocular degeneration. However, aside from neuroprotective paracrine actions, the mechanisms underlying the beneficial effect of MSCs on retinal and corneal tissues are largely unknown. In this study, we investigated the fate and associated characteristics of mitochondria subjected to intercellular transfer from MSCs to ocular cells.

**Methods:** MSCs were cocultured with corneal endothelial cells (CECs), 661W cells (a photoreceptor cell line) and ARPE-19 cells (a retinal pigment epithelium cell line). Immunofluorescence, fluorescence activated cell sorting and confocal microscopy imaging were employed to investigate the traits of intercellular mitochondrial transfer and the fate of transferred mitochondria. The oxygen consumption rate of recipient cells was measured to investigate the effect of intercellular mitochondrial transfer. Transcriptome analysis was performed to investigate the expression of metabolic genes in recipient cells with donated mitochondria.

**Results:** Mitochondrial transport is a ubiquitous intercellular mechanism between MSCs and various ocular cells, including the corneal endothelium, retinal pigmented epithelium, and photoreceptors. Additionally, our results indicate that the donation process depends on F-actin-based tunneling nanotubes. Rotenone-pretreated cells that received mitochondria from MSCs displayed increased aerobic capacity and upregulation of mitochondrial genes. Furthermore, living imaging determined the ultimate fate of transferred mitochondria through either degradation by lysosomes or exocytosis as extracellular vesicles.

**Conclusions:** For the first time, we determined the characteristics and fate of mitochondria undergoing intercellular transfer from MSCs to various ocular cells through F-actin-based tunneling nanotubes, helping to characterize MSC-based treatment for ocular tissue regeneration.

## Introduction

Loss of metabolic homeostasis has been implicated as a common mechanism of degeneration 1. Mitochondria are powerhouses that produce ATP, which is the major source of energy for most cell types. On the basis of this crucial role in energy production, mitochondrial disorders preferentially affect tissues with high energy requirements, such as retina and corneal endothelium in human eyes 2, 3. Primary mitochondrial diseases in human eyes are predisposed by mutations in nuclear DNA or mitochondrial DNA 4, such as Fuchs endothelial dystrophy 5, Leber hereditary optic neuropathy 6, and autosomal dominant optic atrophy 7. In addition to mitochondrial defects caused by genetic factors 8, recent studies have shown that mitochondrial damage and deregulated mitochondrial homeostasis play an essential role in the pathogenesis of acquired ocular diseases, such as diabetic retinopathy (DR), age-related macular degeneration (AMD), glaucoma, and keratoconus 9-12. Ferrington et al. reported that mitochondrial damage of retinal pigmented epithelium (RPE) occurred in early AMD, leading to the metabolic change from oxidative phosphorylation to glycolysis 13, 14. This effect results in RPE robbing glucose from neighboring photoreceptors 15. Therefore, metabolic changes in the retina cause a bioenergetic crisis, eventually leading to the cell death of photoreceptors and/or RPE 14.

Despite the improved knowledge of the mechanism and therapeutic development targeting mitochondrial biogenesis, there is still no clinical treatment with satisfactory effects 16-18. Mesenchymal stem cell (MSC)-based treatment has been proposed as a promising strategy for ocular tissue regeneration 19, 20 due to its immune privileges, anti-inflammatory properties, abundant sources and off-the-shelf formats 21. From the clinicaltrial.gov database, at least 20 clinical trials of MSC-based therapy were registered for ocular diseases. However, the mechanism underlying the beneficial effect of MSCs on retinal and corneal tissues has not been elucidated. In 2004, Rustom and colleagues first reported mitochondrial donation *via* a new form of cell-to-cell interaction based on tunneling nanotubes (TNTs) 22. Previously, we discovered that MSCs could donate mitochondria to retinal ganglion cells and corneal epithelial cells, helping to elucidate the mechanism of MSC-based treatment for ocular diseases 23, 24.

Given the essential role of mitochondrial homeostasis in various ocular diseases, we thus hypothesized that intercellular mitochondrial communication occurred between variant ocular cells and MSCs. The aim of this study was to determine whether injured ocular cells can receive metabolite transfer from surrounding healthy cells and whether MSCs are able to provide exogenous mitochondria to ocular cells, including corneal endothelial cells (CECs), 661W (a photoreceptor cell line) and ARPE-19 (a retinal pigment epithelium cell line). We found that the intake of mitochondria through tunneling nanotubes resulted in an improved metabolic function in the recipient ocular cells. In addition, we determined the ultimate fate of transported mitochondria in recipient cells. Furthermore, we provided *in vivo* evidence that the photoreceptor cells received mitochondria from the grafted MSCs. Our findings demonstrate pronounced intercellular transfer of mitochondria from MSCs to corneal endothelium, RPE cells and photoreceptors, providing new insights into the application of MSC-based treatment for ocular tissue regeneration.

## Methods

### Cell culture

Human MSCs were purchased from Nuwacell (Nuwacell, Cat# RC02003, Hefei, China) and cultured in Dulbecco's Modified Eagle Medium (DMEM), 10% fetal bovine serum (FBS), 1% penicillin and streptomycin. 661W (RRID:CVCL_6240) is a cone photoreceptor cell lineage that is derived from mouse retinal tumors 25. We cultured 661W in Dulbecco's modified Eagle's medium (DMEM) with 10% FBS and 1% penicillin and streptomycin. Human corneal endothelial cells (DSMZ, Cat# ACC-646, RRID: CVCL_2064) were cultured as previously reported 26. Cells were cultured with DMEM/F-12, 10% fetal bovine serum (FBS), 0.5% penicillin and streptomycin. ARPE-19 cells (ATCC, Cat# CRL-2302, RRID: CVCL_0145) were grown in DMEM containing 10% FBS and 1% penicillin and streptomycin and were used between passages 3-6. All cell cultures were maintained in a humidified atmosphere of 95% air and 5% CO_2_ at 37°C.

### Cell labeling and tracking

The mitochondrial Cyto-Tracer fuses a cytochrome C oxidase subunit VIII tag to copGFP (Mito-COX8-GFP, SBI, Cat# Cyto102-PA-1, USA), resulting in copGFP labeling of mitochondria. Lentivirus packaging was performed using the Mito-GFP plasmid prepared, and Mito-COX8-GFP lentivirus was transfected into mitochondrial donor cells 27. CellTrace violet (Invitrogen, Cat# C34557, Carlsbad, CA, USA) was used for cytoplasm labeling. Lysosome-RFP (Invitrogen, Cat# C10597, Carlsbad, CA, USA) was used for lysosome labeling. Phalloidin (Thermo Fisher Scientific, Cat# A22287, RRID: AB_2620155), a high-affinity F-actin probe, was used for F-actin staining of fixed cells.

### Establishment of an *in vitro* mitochondrial injury model and coculture system

We treated cells with 0, 1 and 5 μM rotenone (rot) (Sigma, Cat# R8875) for 2 h to investigate the inhibition of rot in mitochondrial function. Then, 5 µM rot was used to induce the mitochondrial injury model. Rot-treated and untreated recipient cells were subjected to coculture with mitochondrial donor cells at a ratio of 1:1. Next, we seeded the mixed cells at a density of 2 × 10^4^/cm^2^ with 1:1 culture medium.

### Assessment of mitochondrial transfer

We observed mitochondrial transfer under a laser scanning confocal microscope (Leica, RRID: SCR_002140). Furthermore, we counted the Mito-COX8-GFP-positive recipient cells per 100 CellTrace violet-positive recipient cells (n > 5) to quantitatively determine the rate of mitochondrial transfer. In short, cells labeled with CellTrace violet and Mito-COX8-GFP were subjected to coculture at a 1:1 ratio for 24 h. Laser scanning was employed to obtain images under a Leica confocal microscope with an argon laser at a wavelength of 488 nm and a laser at 405 nm. We then used ImageJ software (ImageJ, RRID: SCR_003070) to perform data analysis.

### OCR measurement

To evaluate the mitochondrial function of cells, we used seahorse XFp analyzer (Agilent Technologies, RRID: SCR_013575). The seahorse XFp analysis platform monitors changes in the dissolved oxygen and free protons around the cells, reflecting mitochondrial oxidative phosphorylation for understanding cellular bioenergetics in living cells. We seeded 15,000 CECs in each well of an XF cell culture microplate with 180 µL culture medium and incubated them overnight at 37°C in 5% CO^2^. The culture medium was replaced with 180 µL of XF Basal medium with 17 mM glucose, 0.6 mM pyruvate and 6.98 mM L-glutamine. As specified earlier 24, the determination of the bioenergetic profile was carried out by four measurements: (i) basal respiration - in assay medium with pyruvate (0.6 mM), L-glutamine (6.98 mM), and D-glucose (5.78 mM); (ii) postinhibition ATP synthase activity - by 1.5 µM oligomycin, as well as respiration-driving proton leak and ATP synthesis turnover; (iii) maximal mitochondrial respiratory capacity - following treatment with the uncoupling agent 0.5 µM FCCP (carbonyl cyanide p-trifluoromethoxyphenylhydrazone); and (iv) nonmitochondrial respiration - posttreatment with 2 µM complex I inhibitor, rot, and complex III inhibitor, antimycin A (AA).

### Subretinal injection

All experimental protocols and animal handling procedures were approved by the Faculty Committee on the Use of Live Animals in Teaching and Research of the university. To evaluate mitochondrial transfer from MSCs to retinas *in vivo*, GFP-MSC treatment was performed *via* subretinal injection. GFP-MSCs and PBS were injected into the subretinal space of C57BL/6J mice (IMSR Cat# JAX:000664, RRID: IMSR_JAX:000664) at 4 weeks postnatal. Under anesthesia (100 mg/kg ketamine and 20 mg/kg xylazine, *i.p.* injection), a cell suspension containing 1 × 10^4^ GFP-MSCs in 0.5 µL PBS was slowly injected into the subretinal space of the mice. At 3 days posttransplantation, mice were sacrificed for examination.

### Transcriptome Analysis

Total RNA was extracted from mitochondrial recipient cells using TRIzol reagent (Invitrogen, Cat# 15596018, USA) and the RNeasy Mini Kit (Qiagen, Cat# 74104, USA) following the manufacturer's instructions. The RNA concentration was determined with a Nanodrop 2000 (Thermo Scientific, RRID: SCR_018042). A total quantity of 5 µg of RNA per sample from the CEC, rot-CEC and rot-CEC (MSCs) (cocultured with MSCs and sorted) was used for analysis. RNA-seq data were analyzed using BMKCloud as previously reported 28, 29. Briefly, the Illumina library (Biomarker Technologies, Beijing, China) was used to generate sequencing libraries following the manufacturer's recommendations. Using the HiSeq PE Cluster Kit v4 cBot, clustering of the index-coded samples was performed. Raw data in FASTQ format were processed. Then, clean data were obtained from raw data after removing reads containing adapter poly-N and low-quality reads.

### Statistical analysis

GraphPad software (GraphPad Prism Version 5.03, RRID:SCR_002798) was used for statistical analysis. All values are expressed as the mean ± SD. One-way ANOVA was adopted for comparisons between more than two groups. Student's *t*-test was utilized for two-group comparisons. Differences were considered to be significant at* P* < 0.05.

## Results

### Mitochondria transfer from MSCs to corneal endothelial cells

**Figure 1:**
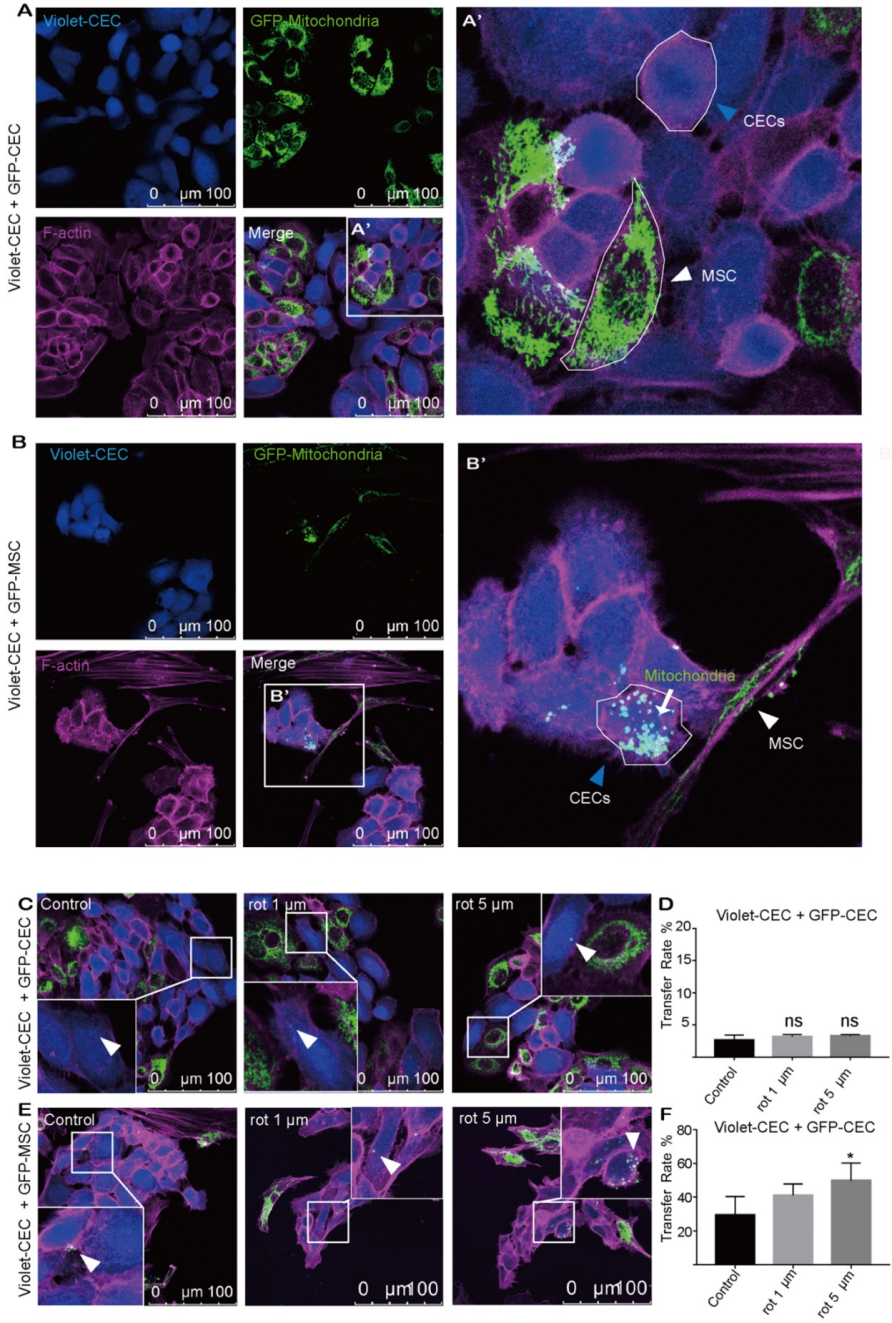
** Mitochondria transfer from MSCs to corneal endothelial cells. A.** Coculture of Mito-COX8-GFP-labeled CECs and violet-labeled CECs; (A') magnified image of box-A'. Arrowheads represent CEC and MSC. **B.** Coculture of GFP-MSCs and violet-CECs; (B') magnified image of box-B'. MSC-derived mitochondria (arrow) in recipient CECs (blue arrowheads). **C-D.** Representative images and intercellular mitochondrial transfer rate from GFP-CECs to violet-CECs that were pretreated with 0, 1 µm, and 5 µm rot. One-way ANOVA, mean ± SD, ns: no significant difference, n = 5. **E-F.** Representative images and rate of mitochondrial transfer from GFP-MSCs to violet-CECs that were pretreated with 0, 1 µm and 5 µm rot. One-way ANOVA, mean ± SD, * P < 0.05, n = 5. rot: rotenone.

To explore whether mitochondria were transferred from CECs or MSCs to nearby CECs, CECs were cocultured with CECs or MSCs under coculture conditions. To track mitochondrial movement, we expressed Mito-COX8-GFP in MSCs (GFP-MSCs) and CECs (GFP-CECs) separately (Figure S1A-C). Then, we labeled CECs with CellTrace violet (violet-CEC), which is illustrated in Figure S1. After 24 h of coculture, GFP-labeled mitochondria from CECs were detected in several of the violet CECs (Figure 1A, D). Interestingly, in coculture experiments using GFP-MSCs as donors, Mito-COX8-GFP-labeled mitochondria were frequently present in 29.42% of the CECs (Figure 1B, F). These results indicate that CECs prefer to obtain 'foreign mitochondrial aid' from MSCs, rather than their CEC neighbors.

To further test the hypothesis that mitochondrial damage of recipient cells causes enhanced mitochondrial uptake, rot, a mitochondrial complex I inhibitor, was applied to induce mitochondrial dysfunction in recipient cells. However, no significant increase in mitochondrial transfer was observed between violet-CEC and GFP-CEC (Figure 1C, D) after pretreatment of violet-CEC with 1 μM or 5 μM rot. As expected, the pretreatment of CECs with both 1 μM and 5 μM rot promoted the mitochondrial uptake of violet-CECs from GFP-MSCs (Figure 1E, F). These data suggest that partial deprivation of mitochondrial function in CECs increased their uptake of mitochondria from MSCs but not from CECs.

### MSCs are active mitochondrial donors for various cell types

**Figure 2:**
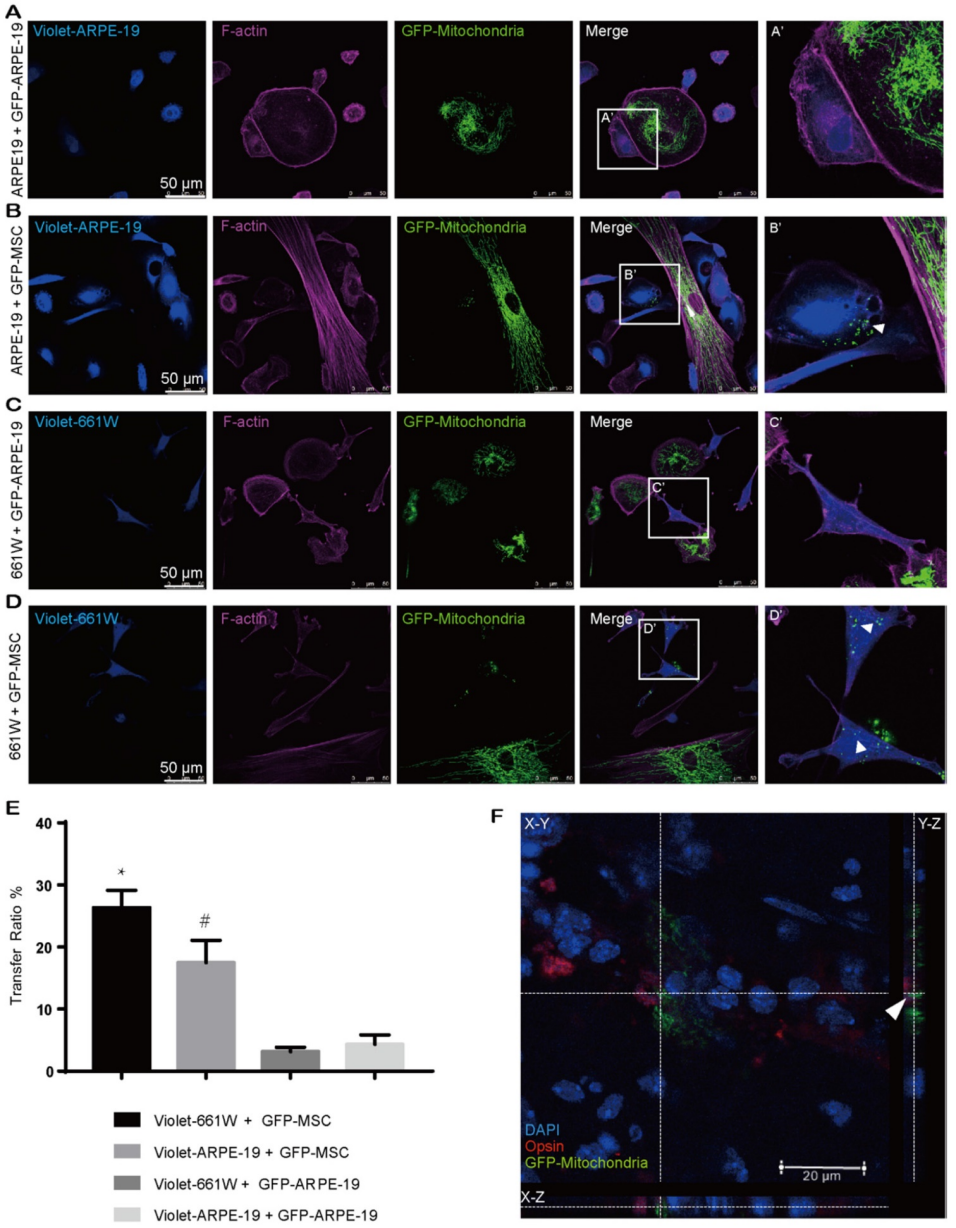
** MSCs are active mitochondrial donors for various cell types. A-D.** Representative images of intercellular mitochondrial transfer between (A) GFP-ARPE-19 and violet-ARPE-19, (B) GFP-MSCs and violet-ARPE-19, (C) GFP-ARPE-19, and violet-661W, (D) GFP-MSCs and violet-661W. (A'-D') Magnified images of box A'-D'. The arrowhead indicates the mitochondria from donors within recipient cells. **E.** Intercellular mitochondrial transfer rate. One-way ANOVA, mean ± SD, * P < 0.05 v.s. violet-661W + GFP-ARPE-19, ^#^* P* < 0.05 v.s. violet-ARPE-19 + GFP-ARPE-19, n=5.** F.** GFP-labeled MSC-transplanted retinas were stained with opsin (red/green cone, red). Mito-COX8-GFP is colocalized with opsin (arrow). Scale bars: 20 µm.

Our abovementioned findings showed that GFP-MSCs had a higher capacity than GFP-CECs to donate mitochondria into violet-CECs. Therefore, we next asked whether the effectiveness of MSCs as an excellent donor was a common event. CellTrace violet-stained ARPE-19 cells were cocultured with Mito-COX8-GFP tagged ARPE-19 (GFP-ARPE-19) cells and GFP-MSCs. Rare transfer of mitochondria was detected in violet-ARPE-19 cells when they were cocultured with GFP-ARPE-19 cells (Figure 2A, E). However, a significant increase in mitochondrial GFP fluorescence in violet-positive ARPE-19 cells was obtained in the cocultivation of violet-ARPE-19 cells and GFP-MSCs for 24 h (Figure 2B, E). Similarly, the photoreceptor cell line 661W had a higher affinity to accept mitochondria from cocultured GFP-MSCs than GFP-ARPE-19 cells (Figure 2C-E). Taken together, these results demonstrate that MSCs are efficient mitochondrial donors to various ocular cells, while healthy ocular cells have a limited capacity for mitochondrial donation.

To determine whether MSCs could transfer mitochondria to photoreceptors *in vivo*, we performed subretinal transplantation of GFP-labeled MSCs in C57BL/6J mice. In the 3-day posttransplanted retina, Mito-COX8-GFP signals were detected in the mouse photoreceptors (Figure 2F, Video S1), indicating that the photoreceptors directly received mitochondria from the injected MSCs.

### Mitochondria are transported to neighboring cells *via* intercellular tunneling nanotubes

Next, we investigated how the mitochondria were transported from donor to recipient cells. Using time-lapse live imaging, we investigated the dynamic state of mitochondrial translocation upon cocultivation of Mito-COX8-GFP-labeled MSCs and CECs. Remarkably, MSC-derived mitochondria streaming toward the CECs were observed (Video S2). However, it was not apparent whether mitochondrial transfer occurred through direct transfer of cytoplasmic mitochondria or merely discretion of mitochondria. To answer this question, we separated CellTrace red-CECs with MSCs in a Transwell system. Intriguingly, we found that physical contact but not exocytosis was a prerequisite for mitochondrial transport (Figure S2).

**Figure 3:**
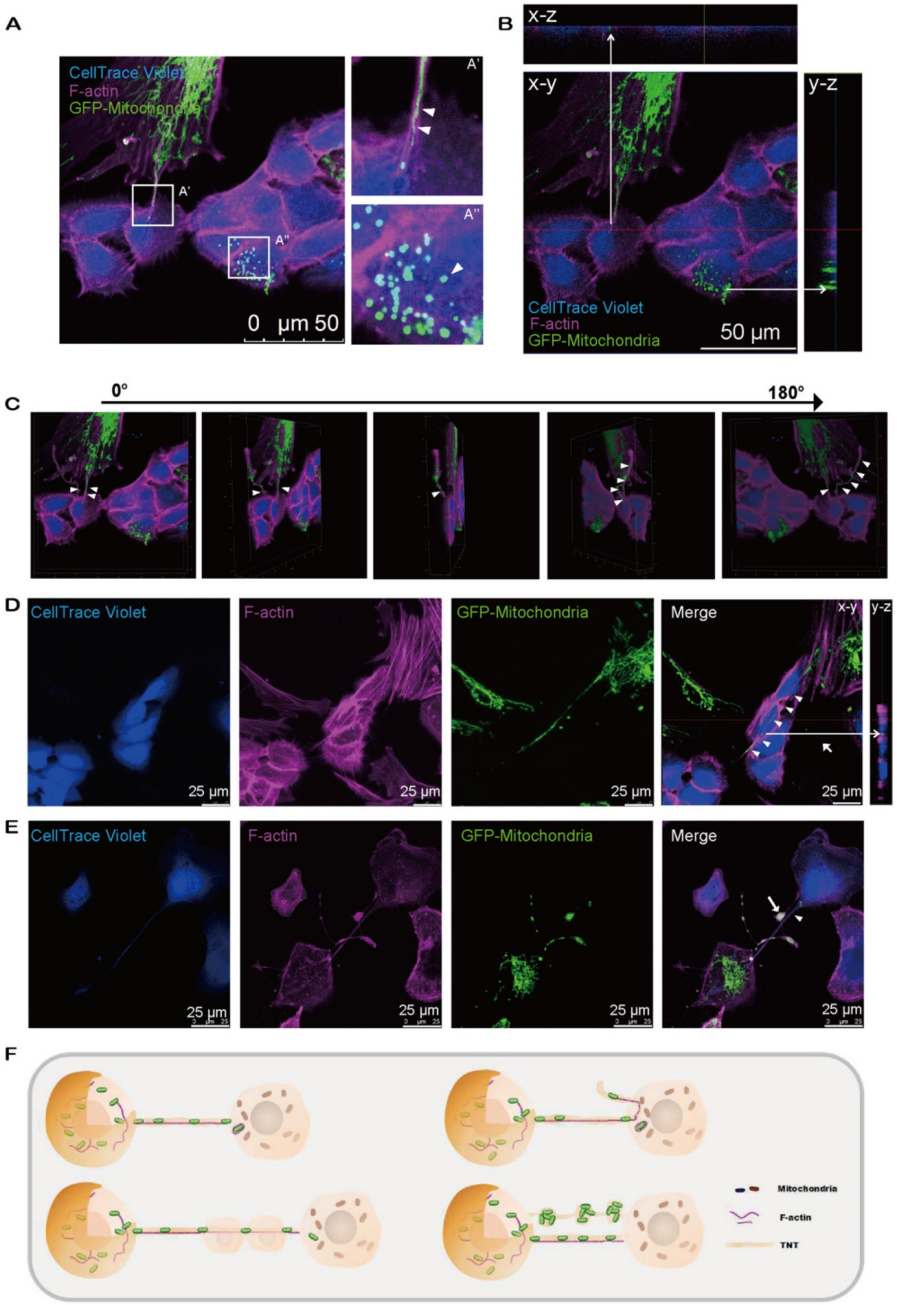
** Intercellular mitochondrial transfer *via* TNTs. A.** Mito-COX8-GFP signals were observed in violet CECs at 24 h after cocultivation. **(A')** Magnified image of box-A'. Mitochondria transferred from GFP-MSCs to violet-CECs by tunneling nanotubes (TNT) (arrowheads). **(A'')** Magnified image of box-A''. Arrowhead displays the mitochondria from GFP-MSCs within violet-CECs. **B.** Z-stack images display the Mito-COX8-GFP signal localized within TNTs and cytoplasm of CECs. **C.** The 180-rotated 3D synthesized images show that the TNT has a floating structure. Arrowheads show mitochondria within floating TNT. See related Video S3. **D.** Mitochondria (arrowhead) from donor cells were transferred to recipient cells *via* F-actin-positive TNTs across several intermediate cells. Z-stack imaging demonstrated that the Mito-COX8-GFP signals were surrounded by F-actin signals within the cytoplasm of CECs (arrow). **E.** GFP-mitochondria penetrated through TNTs individually (arrowhead) or in groups (arrow). **F.** The schematic diagram shows the key properties of TNT-based mitochondrial transfer.

We next observed the relocation of mitochondria from MSCs to CECs through the F-actin-positive tubular microstructure, tunnel nanotube (TNT), which enabled the consecutive passage of mitochondria (Figure 3A'). Transferred mitochondria were detected in CECs (Figure 3A''), illustrating the translocation of mito-GFP-labeled mitochondria from MSCs to CECs (Figure 3B). Furthermore, cytochalasin B (CB), an inhibitor of actin polymerization and the interaction of actin, reduced the mitochondrial transfer rate (Figure S3). We found that the TNTs hovered in the culture medium but did not contact the substrate (Figure 3C; see also Video S3). Interestingly, we observed that the F-actin-positive TNTs, in some cases, pass through several cells internally to reach their target cells to transfer mitochondria (Figure 3D). In addition, the transferred mitochondria were observed to be either lining up one by one through TNTs (Figure 3A'; Figure 3E) or being transferred in clusters of several mitochondria and squeezing through the nanotubes (Figure 3E).

### Mitochondrial “donation” improved energy metabolism in recipient cells

**Figure 4:**
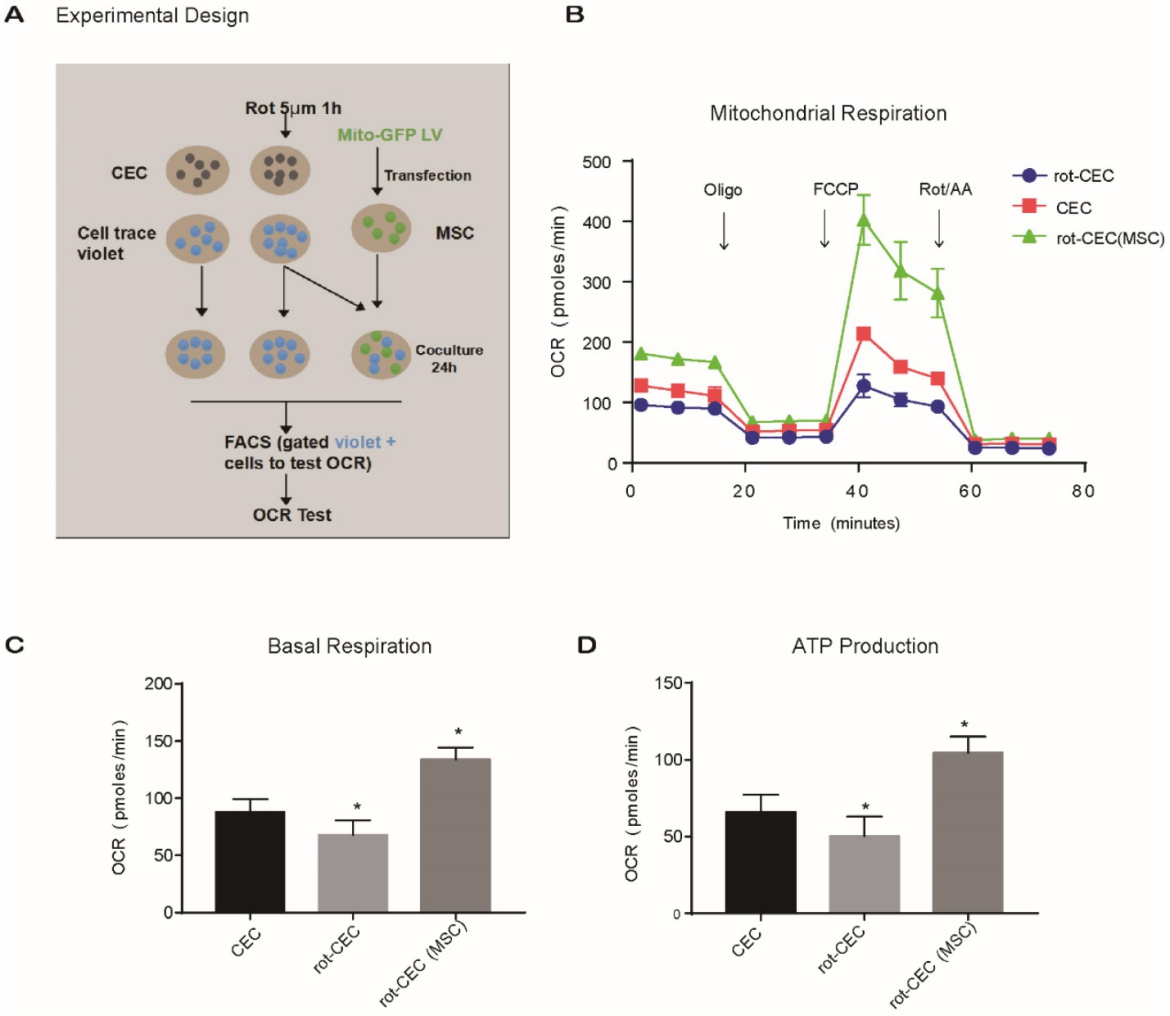
** Mitochondrial internalization improved the bioenergetic profile in recipient cells. A**. Experimental design of the oxygen consumption rate (OCR) using FACS-sorted CEC, rot-CEC, and rot-CEC (MSC). **B.** A total of 15 OCR measurements were performed over a 2 h period of basal respiration, oligomycin-sensitive respiration, maximal respiratory capacity and nonmitochondrial respiration. The data are presented as the mean ± SD. **C-D.** Basal respiration and ATP production of CECs. One-way ANOVA, mean ± SD, *P < 0.05 v.s. CECs, n = 4. rot: rotenone.

To investigate the effect of intercellular mitochondrial transfer on the energy metabolism of recipient cells, we examined the oxygen consumption rate (OCR) of CECs using an extracellular flux analyzer. We compared the mitochondrial respiratory function of normal CECs, rot-pretreated CECs (rot-CECs), and rot-CECs after coculture with MSCs (Figure 4A). First, we determined that 5 μM rot treatment significantly reduced the basal respiratory capacity and ATP production of CECs (Figure 4B-D). Surprisingly, after coculture with MSCs, rot-CECs displayed a significant increase in basal respiration and ATP production (Figure 4B-D). This result suggests that mitochondrial donation from MSCs reverses mitochondrial impairment in CECs after rot treatment.

**Figure 5:**
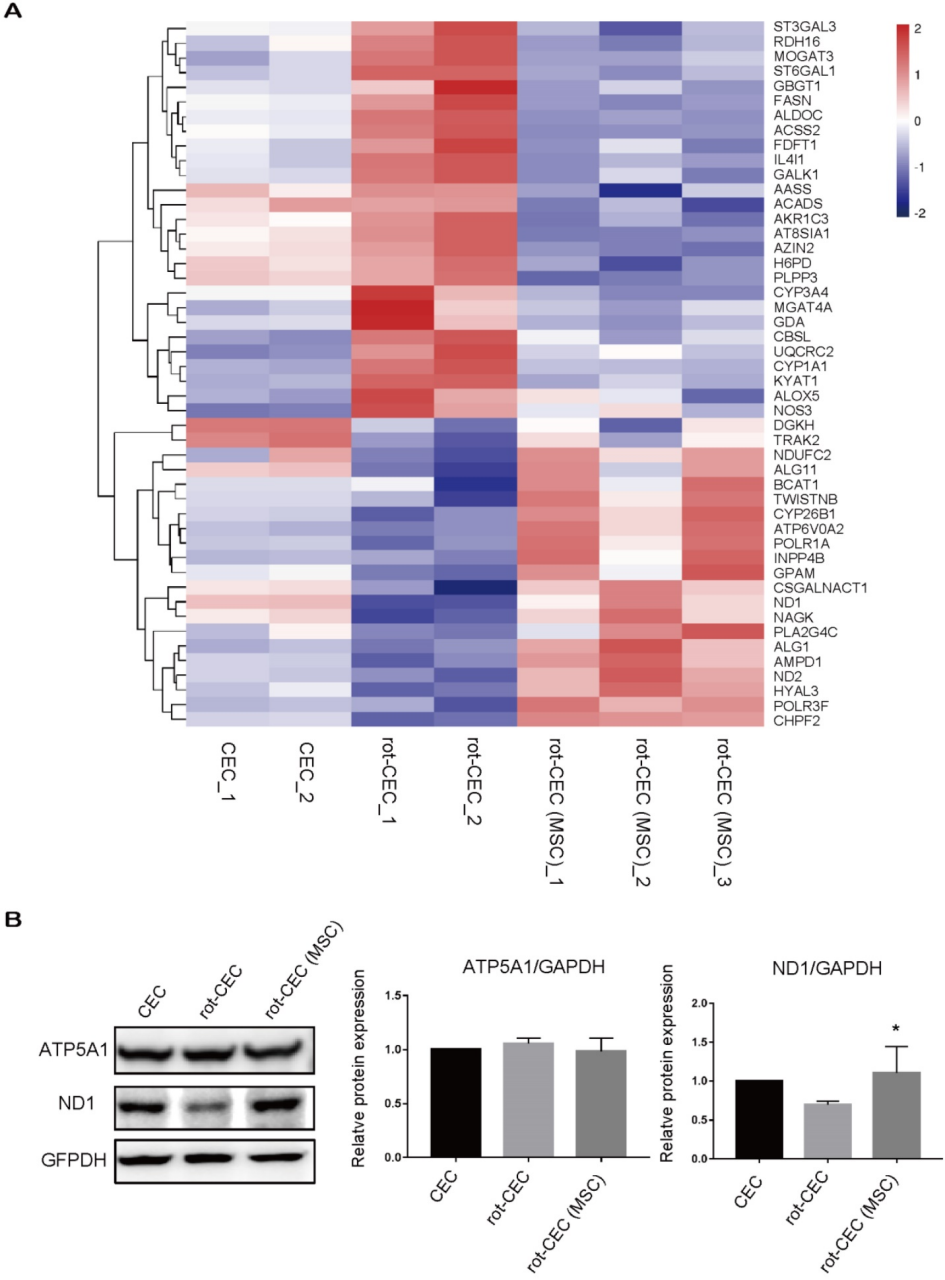
** Internalization of exogenous mitochondria altered gene and protein expression patterns. A.** Hierarchical clustering of metabolic genes. Data are presented as fragments per kilobase of transcript per million mapped reads (FPKM). Red: upregulated expression; Blue: downregulated expression. Validation of the CEC identity by sequencing analysis of signature genes is shown in Figure S4. **B.** Protein electrophoresis and quantification of ATP5A1 and ND1. One-way ANOVA, mean ± SD, *P< 0.05 v.s. rot-CEC, n = 3. Abbreviations; ATP5F1A: ATP Synthase F1 Subunit Alpha; ND1: mitochondrially encoded NADH ubiquinone oxidoreductase core subunit 1; rot: rotenone.

We next investigated the expression in recipient cells with or without donated mitochondria. The top pathway enrichment term of differentially expressed intersecting mRNAs was metabolic pathways (Figure S4). By investigating the metabolic gene expression in rot-pretreated CECs, we found increased expression of genes involved in glycolysis (H6PD and ALDOC), lipid metabolism (ACADS, PLPP3, FASN, and GBGT1), and protein metabolism (ST3GAL3, GBGT1, and MGAT4A). Mitochondrial components, including mitochondrial respiratory chain complex I, the mitochondrial membrane, and the mitochondrial matrix component (BCAT1, NDUFC2, ND2, ND1, and GPAM), were significantly decreased (Figure 5A). In contrast, mitochondrial uptake in rot-CECs led to an opposite transcriptomic change in these genes (Figure 5A), suggesting that mitochondrial transfer from MSCs reversed the alteration of metabolic gene expression of mitochondrial damaged CECs *in vitro.* The protein expression level was detected by western blotting. The protein level of ND1 was decreased when pretreated with rot in CECs and was upregulated after cocultivation with MSCs (Figure 5B).

A line of evidence has shown that MSC-based tissue repair might be driven by paracrine factors from MSCs 30-32. Therefore, we compared the proliferative capacity between rot-CECs or CECs with conditional MSC medium and transferred mitochondria. As a result, conditioned medium from MSCs did not change the proliferation potential in rot-CECs, while MSC-derived mitochondria transported into rot-CECs significantly enhanced their proliferative capacity (Figure S5). These results again support that mitochondrial “donation” improved energy metabolism in recipient cells.

### Ending fate of the internalized mitochondria in the recipient cells

**Figure 6:**
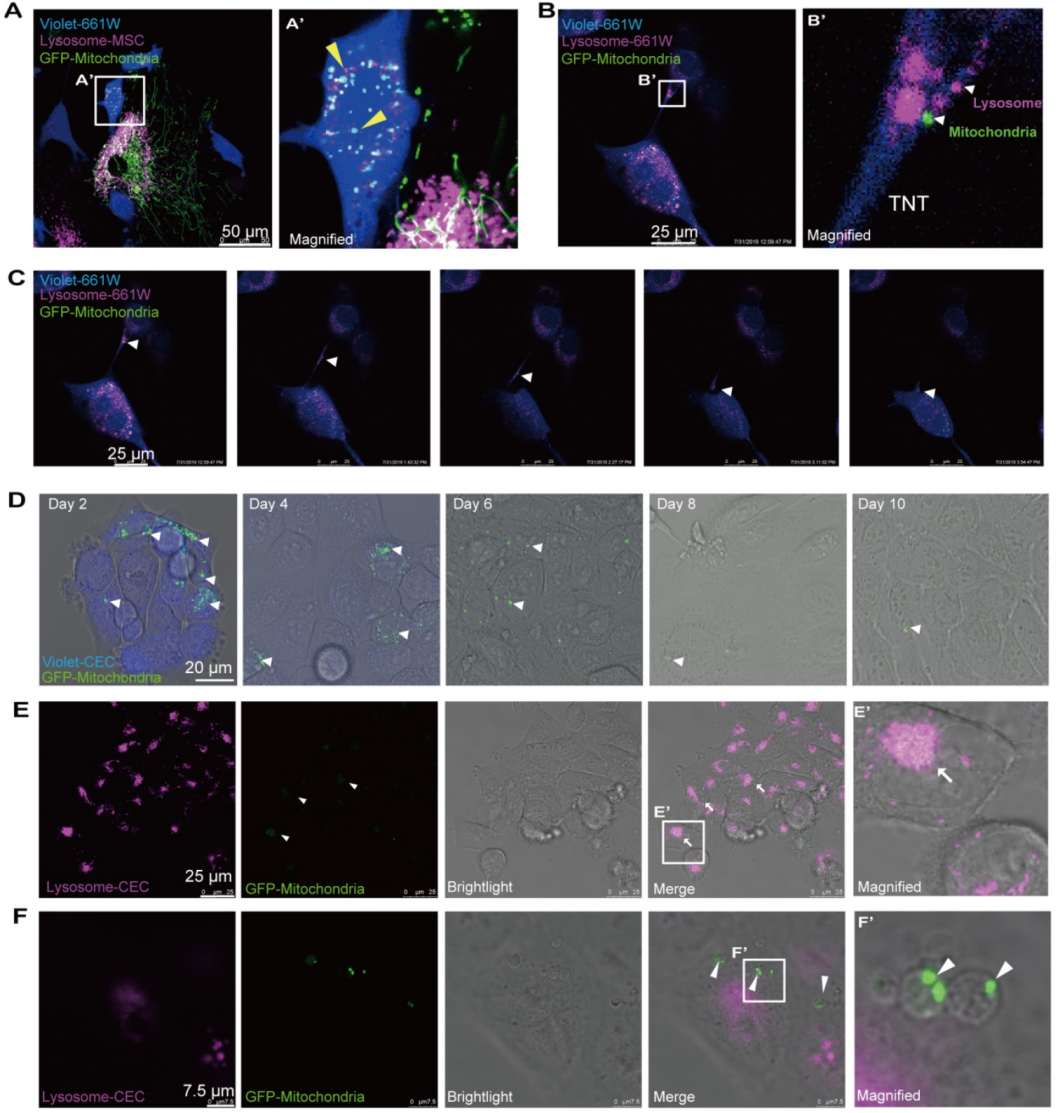
** Ending fate of the internalized mitochondria in the recipient cells. A.** Time-lapse image and magnified image (A') show the transferred mitochondria and lysosomes (arrowheads) within recipient cells. **B.** Image and magnified image (B') show mitochondria (green, arrowhead) transferred from MSCs, and lysosomes (fuchsia, arrowheads) of recipient cells were transported to other recipient cells by tunneling nanotubes (TNTs). **C.** TNT links one cell with another. Arrowheads in the inset point to the movement of mitochondria and lysosomes in the tunnel. See related Video S5. **D.** GFP-mitochondria signals in FACS-sorted recipient cells at days 2, 4, 6, 8, and 10 after cocultivation; arrowheads indicate the mitochondria within the recipient cells. **E.** Colocalization of weak GFP-mitochondrial signals (arrowheads) and host lysosomes (arrows) in recipient cells at day 8. **F.** Some transferred mitochondria were found in a circular bubble-like vesicle (arrowheads) at day 8.

Next, we attempted to determine the ultimate fate of the internalized mitochondria in the recipient cells. To track the fate of the transferred mitochondria, we labeled the mitochondria with GFP and lysosomes with Cyto-Tracer Red in donor MSCs that were cocultured with violet-tagged 661W photoreceptor cells. Interestingly, we observed that mitochondrial transfer was coupled with lysosomes through the TNTs from MSCs to 661W cells (Figure 6A). No significant fluorescence colocalization of the transferred mitochondria and lysosomes was observed in the recipient cells within 48 h after cocultivation (Figure 6A), implying that the transferred mitochondria might not be degraded by the lysosomes moved from MSCs.

Then, we investigated whether the transferred mitochondria were degraded by the lysosomes of recipient cells. The live imaging of host lysosomes tagged with lysosome Cyto-Tracer and MSC-derived mitochondria in the recipient cells (Video S4 and Figure S6) clearly showed that the distribution of donor mitochondria from MSCs was independent of the host lysosomes in recipient cells (Pearson's r = 0.025), implying that the host lysosome did not digest and decompose the transferred mitochondria within 48 h. Interestingly, the transferred mitochondria (from MSCs) and the host lysosome of 661W could be transported from 661W to 661W *via* TNT (Video 4 and Figure 6B-C).

Next, we attempted to determine the fate of internalized foreign mitochondria in recipient cells in longer terms. The cells were thereafter photographed on the second, fourth, sixth, eighth, and tenth days after cocultivation. Over time, the GFP-tagged mitochondria in the recipient cells gradually weakened (Figure 6D). The GFP signals initially appeared as dot-like clusters and then turned into sheet-like signals that overlapped with the host lysosome around day 8 (Figure 6E). In addition to the digested foreign mitochondria, we found that the GFP-positive mitochondria were gradually packaged in 3-5 μm round bubbles and excreted to extracellular vesicles at and after day 8 (Figure 6F, Video S6). These data demonstrate that the transferred mitochondria were either digested by the host lysosomes or excreted outside after 8 days.

## Discussion

MSCs have been considered a promising cell source for regenerating corneal tissues and retina 20, 33. However, a number of studies have shown that the therapeutic effect of MSC-based treatment might be ascribed to anti-inflammatory and paracrine effects, rather than cell replacement 34, 35. A recent study reported mitochondrial donation as a key role of MSC-based therapy 36. Our previous study also demonstrated that intercellular mitochondrial transport is a vital mechanism for the regeneration of corneal epithelial cells and retinal ganglion cells 23, 24. In this study, we described intercellular mitochondrial transportation from MSCs to corneal endothelial cells, photoreceptors, and retinal pigment cells. Importantly, recipient cells that received mitochondria from MSCs exhibited increased mitochondrial respiratory abilities and elevated expression of mitochondrial structure- and function-related genes. In addition, we found the ultimate fate of transferred mitochondria in the recipient cells.

A line of evidence has shown that mitochondrial dysfunction is critical in the pathogenesis of various retinal and corneal diseases 37-39. In diabetic retinopathy, increased mitochondrial fission and increased damage to mitochondrial DNA in the retinal vasculature precede apoptosis of retinal endothelial cells 40, 41. In AMD, RPE cells with dysfunctional mitochondria begin to utilize glucose to generate ATP *via* glycolysis, thereby stealing glucose from the photoreceptors, eventually leading to the cell death of photoreceptors or/and RPE 14. Therefore, targeting mitochondrial dysfunction may be an approach to prevent the development and progression of both retinal and corneal degeneration. In this study, we found that rot-pretreated cells that received mitochondria from MSCs exhibited enhanced mitochondrial function (Figure 4B-D). Elevated expression of mitochondrial structure and function-related genes in recipient cells with transferred mitochondria further indicated the effect of intercellular mitochondrial transport (Figure 5). These results are consistent with previous studies showing that intercellular mitochondrial transport increased the aerobic capacity of cells with mitochondrial disorders 42.

Our results clearly indicated intercellular mitochondria transfer in a cell type-dependent manner. Mitochondria were rarely transferred between intergroup corneal endothelial cells (Figure 1A, D), RPE cells (Figure 2A, E), or intragroup photoreceptors and RPE cells (Figure 2C, E). In contrast, MSCs are an open-handed mitochondrial donor for various ocular cell types (Figure 1-2). The difference in mitochondrial donation capacity has been proposed to be linked to mitochondrial quality and quantity, as well as the expression of mitochondrial motor proteins. MSCs with mitochondrial defects lost their rescue capability under cocultivation conditions 24. Swati et al*.* indicated that the bioenergetics of donor mitochondria may be a critical factor in regulating differential mitochondrial transfer 43. Ahmad et al*.* and Zhang et al*.* suggested that the effective mitochondrial donation of MSCs might be related to the high expression of intrinsic Rho GTPase 1 (MIRO1) 36, 44.

In the present study, we found that direct contact is a prerequisite for TNT formation (Figure S3) and identified F-actin-based TNTs bridging MSCs and recipient cells (Figure 3). This finding is consistent with previous studies showing that F-actin-based TNTs mediate mitochondrial transfer between human MSCs and other cells 44, 45. Moreover, a significantly reduced mitochondrial transfer rate by CB and high expression of F-actin-related genes, including CAP2, NEXN, and ACTN1 (Figure S3), indicated that F-actin expression might be related to intercellular mitochondrial transfer. Notably, our results indicated that F-actin-based TNTs could pass through several nonobjected cells and reach the target cells (Figure 3D). More interestingly, the mitochondrial recipient cells could second transmit the received mitochondria to other cells afterward (Figure 6B, C). These findings expand the current understanding of TNT-based intercellular transfer of cellular organelles.

The destiny of the translocated mitochondrion in the recipient cells has not been elucidated. In this study, we revealed that the transferred mitochondria were either degraded by the coupled lysosomes or host lysosome in the recipient cells (Figure 6A-C and Video S4) or excreted outside in vesicles (Figure 6F, Video S6). Johnson et al. discovered that extracellular vesicles containing intact organelles excreted by acute lymphoblastic leukemia cells, internalized by other cells, thereafter led to phenotypic transformation 46. However, in our study, we did not observe that the mitochondria-containing vesicles were internalized again by other recipient cells, indicating a variety of ultimate fates for the transferred organelles.

In summary, we reported the trait of intercellular mitochondrial communication between intergroup ocular cells and mesenchymal stem cells to various ocular cells. We revealed that the donation process depends on the F-actin-based membranous channel and that tunneling nanotubes and donated mitochondria lead to an improved metabolic function in recipient ocular cells. These findings provide novel insights into organelle donation from MSCs to ocular cells and their potential advantages for new strategies of stem cell-based therapy.

## Abbreviations

AA: antimycin A
ACADS: acyl-CoA dehydrogenase short chain
ACTN1: actinin alpha 1
ALDOC: aldolase, fructose-bisphosphate C
AMD: age-related macular degeneration
ATP5F1A: ATP Synthase F1 Subunit Alpha
BCAT1: branched chain amino acid transaminase 1
CAP2: cyclase associated actin cytoskeleton regulatory protein 2
CB: cytochalasin B
CEC: corneal endothelial cell
COX8: cytochrome c oxidase subunit VIII
DR: diabetic retinopathy
FASN: fatty acid synthase
FBS: fetal bovine serum
FCCP: carbonyl cyanide p-trifluoromethoxyphenylhydrazone
GBGT1: globoside alpha-1,3-N-acetylgalactosaminyltransferase 1
GBGT1: globoside alpha-1,3-N-acetylgalactosaminyltransferase 1
GPAM: glycerol-3-phosphate acyltransferase, mitochondrial
H6PD: hexose-6-phosphate dehydrogenase/glucose 1-dehydrogenase
MGAT4A: alpha-1,3-mannosyl-glycoprotein 4-beta-N-acetylglucosaminyltransferase A
MSC: mesenchymal stem cell
mtDNA: mitochondrial DNA
ND1: mitochondrially encoded NADH ubiquinone oxidoreductase core subunit 1
ND2: mitochondrially encoded NADH ubiquinone oxidoreductase core subunit 2
nDNA: nuclear DNA
NDUFC2: NADH ubiquinone oxidoreductase subunit C2
NEXN: nexilin F-Actin binding protein
OCR: oxygen consumption rate
PLPP3: phospholipid phosphatase 3
rot: rotenone
RPE: retinal pigmented epithelium
ST3GAL3: ST3 beta-galactoside alpha-2,3-sialyltransferase 3
TNTs: tunneling nanotubes
