# Supplementary material for: Bioenergetic Crosstalk between Mesenchymal Stem Cells and various Ocular Cells through the intercellular trafficking of Mitochondria
Source: Theranostics. 2020 Jun 5;10(16):7260–72. doi: 10.7150/thno.46332 (PMC7330858; doi:10.7150/thno.46332)

Bioenergetic crosstalk between mesenchymal stem cells and various ocular cells through the intercellular trafficking of mitochondria

Dan Jiang[1], Fang-Xuan Chen[1], Heng Zhou[1], Yang-Yan Lu[1], Hua Tan[1], Si-Jian Yu[1], Jing Yuan[1], Hui Liu[1], Wen-Xiang Meng[2], Zi-Bing Jin[1,3*]

[1]Laboratory for Stem Cell and Retinal Regeneration, Institute of Stem Cell Research, Division of Ophthalmic Genetics, the Eye Hospital, Wenzhou Medical University; National Center for International Research in Regenerative Medicine and Neurogenetics, Wenzhou, 325027 China;

[2]State Key Laboratory of Molecular Developmental Biology, Institute of Genetics and Developmental Biology, Chinese Academy of Sciences, Beijing, 100101 China;

[3]Beijing Institute of Ophthalmology, Beijing Tongren Eye Center, Beijing Tongren Hospital, Capital Medical University, Beijing Ophthalmology and Visual Science Key Laboratory, Beijing, 100730 China.

*Correspondence: jinzibing@foxmail.com

Running title: Trait of mitochondrial transfer from MSCs to ocular cells

Methods

Transwell assay

We conducted Transwell assay to evaluate the necessity of physical contact for intercellular mitochondrial transfer. CellTracker red-labeled CECs (CellTracker™ Red CMTPX Dye, Invitrogen, C34552, USA) on the lower chamber of Transwell were co-cultured with 1) normal medium, 2) supernatant of MSCs, 3) MSCs on the upper chamber of Transwell, and 4) MSC on the lower chamber of Transwell respectively. After 24 h co-cultivation, the red-CECs were harvested for FACS sorting and images were taken using the laser scanning confocal microscope.

Flow Cytometry

Mitochondrial recipient cells were prepared for OCR, transcriptome and ending fate of the internalized mitochondria analysis. Briefly, CellTrace violet-labeled cells and Mito-COX8-GFP-labeled MSCs were co-cultured at a ratio of 1:1 in a 6-well plate for 24 h. The positive beads were gated on the FL1-FITC, FL6-Pacific blue plot. Pacific blue and FITC specific fluorescence were assessed.

Immunofluorescence and immunocytochemistry analyses

To obtain images of live cells, we transfected mitochondrial donor cell with Mito-COX8-GFP virus. Before co-culture, we labeled the mitochondrial recipient cells with CellTrace violet or CellTracker red. After fixation, cells were permeated for 5 min with 0.1% Triton X-100, followed by 30 min cultivation with 1% bovine serum albumin. Next, we performed staining of the cells with Alexa Fluor™ 647 phalloidin for 20 min at room temperature. After two-fold washing with PBS, the cells were

air-dried and mounted in a permanent mounting medium.

Evaluation of mitochondrial transfer in whole mount retina

At day 3 after subretinal injection, eyes of mice were enucleated and fixed in 4% PAF (paraformaldehyde) for 30 min at 4 °C followed by a cornea puncture and an extra of 30 min' fixation. Then, the retina was detached carefully from the underlying RPE and sclera. After soaking with 5% BSA and 0.5% Triton-X-100, the retinas were incubated with anti-Opsin antibody (Millipore Cat# AB5405, RRID: AB_177456) at 4 °C overnight followed by secondary antibody and DAPI at room temperature for 2 h. After washing with PBST for 3 times, retina was flatted and mounted. Fluorescent images were taken using the fluorescent microscope.

Figure and video legends

Figure S1. Cell labeling. **A.** lentiviral-mediated mitochondrial-specific fragment fused with green florescence protein (Mito-COX8-GFP) was used to label mitochondria of donors. **B.** MSCs labeled with Mito-COX8-GFP. **C.** CECs labeled with Mito-COX8-GFP. D. CECs labeled with CellTrace violet. Scale bars: 75 μm.

Figure S2. Transwell assay to evaluate the necessity of physical contact for cell-cell mitochondrial transfer. **A-D.** CellTracker red-labeled CECs on the lower chamber of Transwell were co-cultured with A) normal medium, B) supernatant of MSCs, C) MSCs on the upper chamber of Transwell, D) and MSC with physical contact on the lower chamber of Transwell respectively. Mito-COX8-GFP signals were detected in the cytoplasm of the red-CECs in D (arrowhead).Arrow Scale bars: 20 μm.

Figure S3. A. Co-culture of GFP-MSCs and violet-rot-CECs; (A') magnified image of box-A'. Arrow show MSC-derived mitochondria in recipient CECs. **B.** Co-culture of cytochalasin B (CB) pretreated GFP-MSCs and violet-rot-CECs; (B') magnified image of box-B'. **C.** Intercellular mitochondrial transfer rate. **D.** Bar graph shows gene expression of ACTN1, NEXN and CAP2 in rot-CEC (MSC) (fold change of rot-CEC (MSC) vs. rot-CEC). GFP-MSC^{CB}: GFP-MSCpretreated with 350 nM CB for 24 h before coculture; rot: rotenone; rot-CEC^{CB}: rot-CEC pretreated with 350 nM CB for 24 h before coculture.

Figure S4. A. Top 20 pathway enrichment terms for differentially expressed intersection mRNAs, including up-regulated pathway and down-regulated pathway

(rot-CECs v.s. rot-CECs (MSC)). **B.** Hierarchical clustering of CEC signature genes. Data are presented as fragments per kilobase of transcript per million mapped reads (FPKM). Blue: downregulated expression; Red: upregulated expression; rot: rotenone.

Figure S5. Proliferation potential of CECs. Proliferative capacity of FACS-sorted CECs from different groups was evaluated, inculding CECs, rot-CECs, CECs with MSC supernatant, rot-CECs with MSC supernatant and rot-CECs cocultured with MSC. One-way ANOVA, mean ± SD, ns: no significant difference,*$P < 0.05$, n = 4.

Figure S6. Transferred mitochondria and the host lysosomes in the recipient cells. The distribution of host lysosomes (Fuchsia) and MSC-derived mitochondria (Green) in the recipient cells at 48 h after co-cultivation.

Video S1. A video of reconstructed Z-stack images shows MITO-COX8-GFP -tagged mitochondria within opsin positive cone.

Video S2. Camera phase of a Time-Lapse video shows that mitochondria were being transferred from MSCs to CECs.

Video S3. A video of reconstructed Z-stack images shows GFP-MSCs were co-cultured with violet-CECs and GFP-mitochondria were transferring from MSC to CEC *via* a tunneling nanotubes (TNTs) structure.

Video S4. Camera phase of a Time-Lapse video shows the distribution and movement of transferred mitochondria and host lysosomes within violet-CECs.

Video S5. Camera phase of a Time-Lapse video shows the GFP-mitochondria (from

MSCs) and host lysosomes were transferring from one 661w to another.

Video S6. Camera phase of a Time-Lapse video shows the GFP-mitochondria were gradually packaged in 3-5 μm round bubbles and excreted to extracellular vesicles.

Supplemental Figures

Figure S1

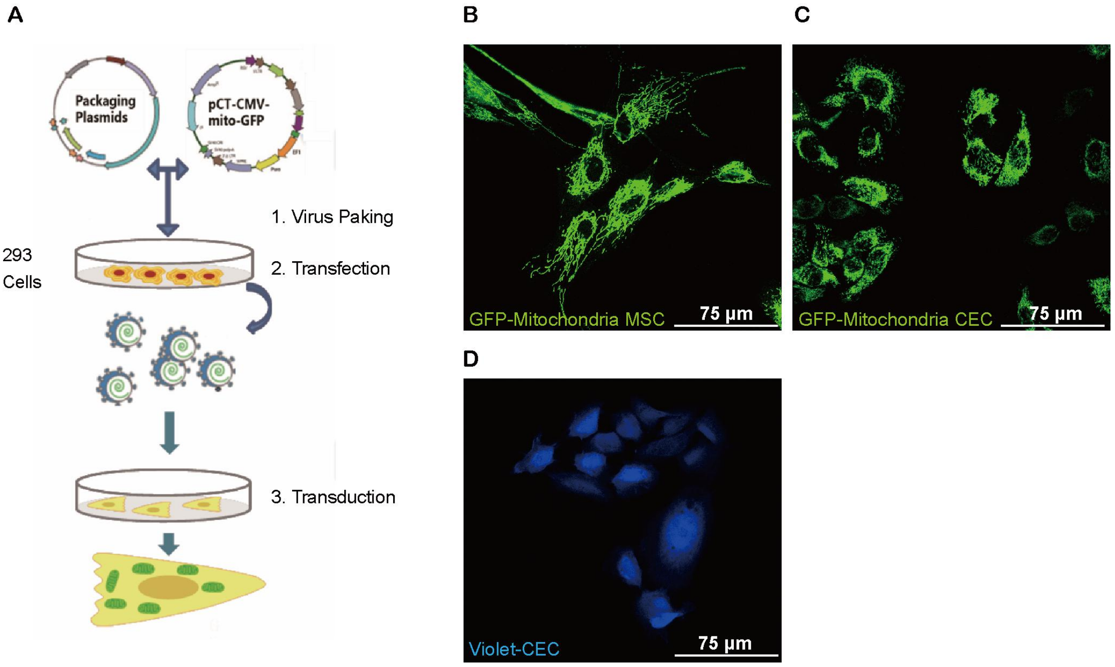

Figure S2

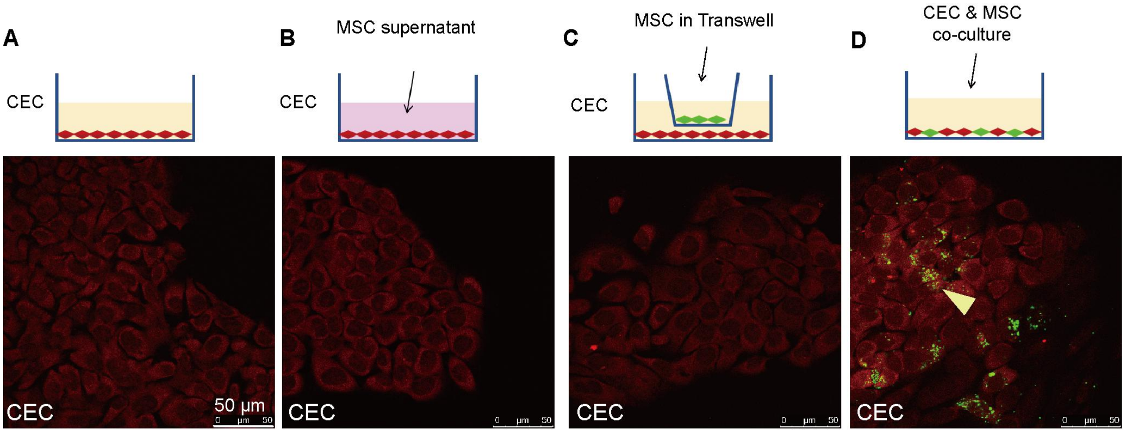

Figure S3

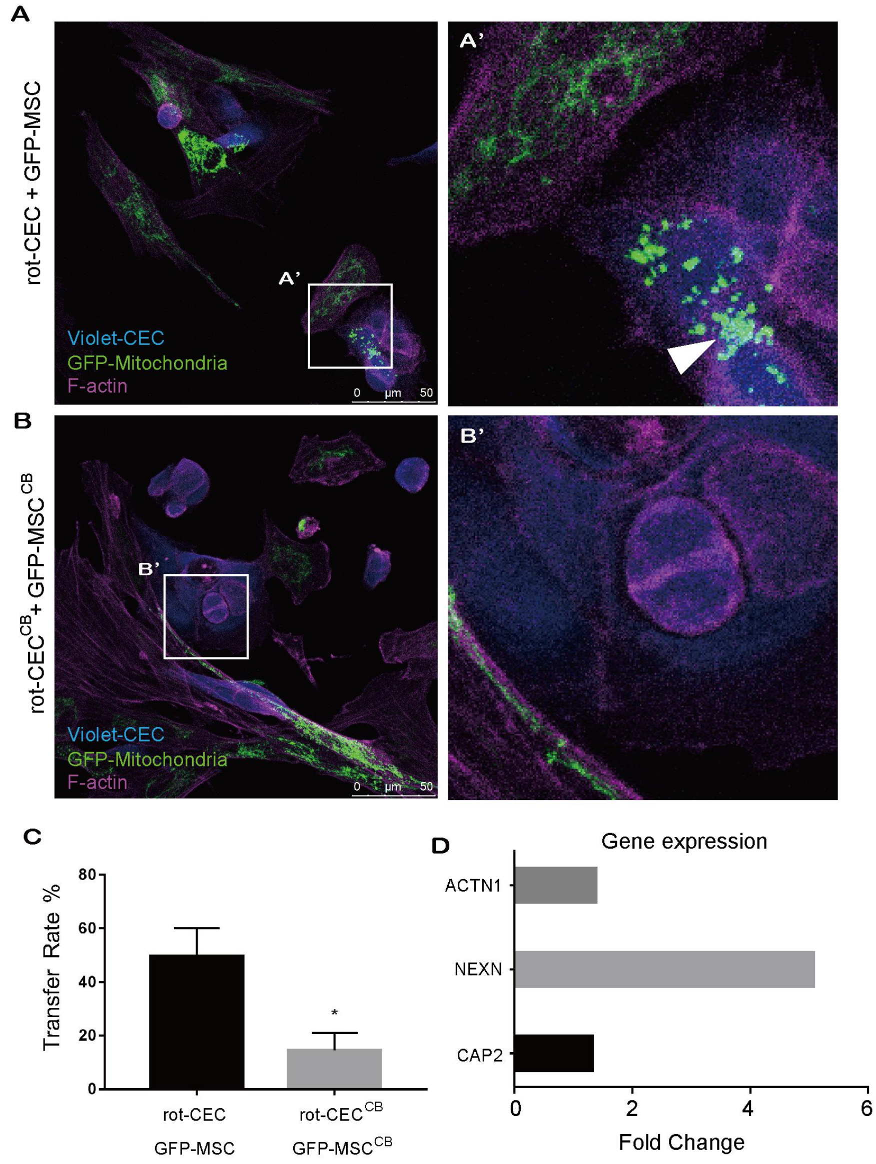
A
rot-CEC + GFP-MSC
A'
Violet-CEC
GFP-Mitochondria
F-actin
0 μm 50
B
rot-CEC^CB + GFP-MSC^CB
B'
Violet-CEC
GFP-Mitochondria
F-actin
0 μm 50
C
Transfer Rate %
80
60
40
20
0
*
rot-CEC
GFP-MSC
rot-CEC^CB
GFP-MSC^CB
D
Gene expression
ACTN1
NEXN
CAP2
0
2
4
6
Fold Change

Figure S4

A

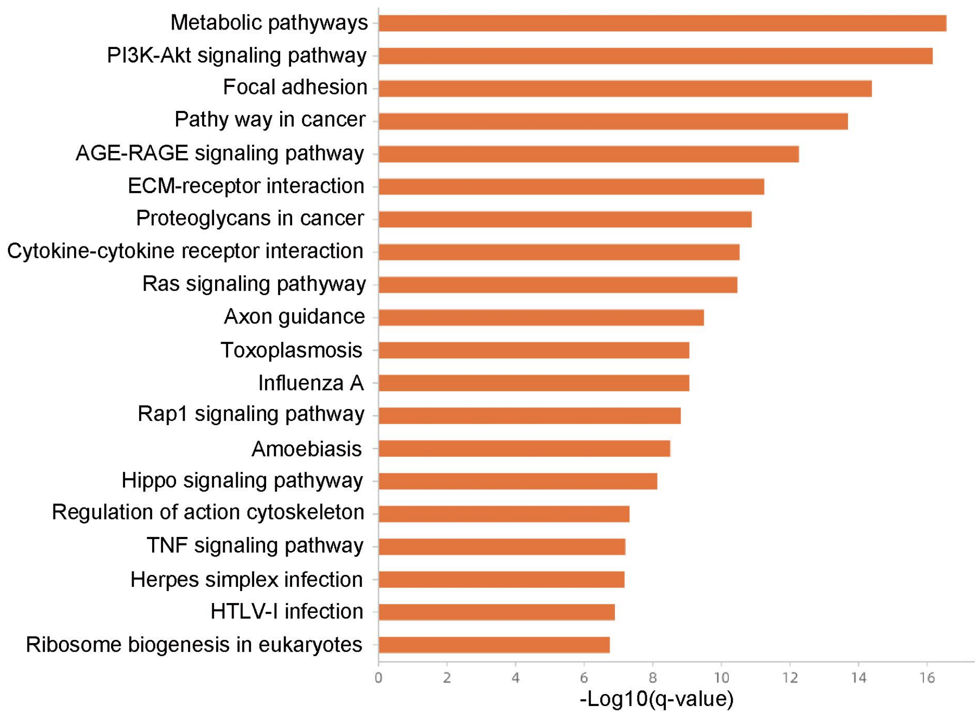
Metabolic pathyways
PI3K-Akt signaling pathway
Focal adhesion
Pathy way in cancer
AGE-RAGE signaling pathway
ECM-receptor interaction
Proteoglycans in cancer
Cytokine-cytokine receptor interaction
Ras signaling pathyway
Axon guidance
Toxoplasmosis
Influenza A
Rap1 signaling pathway
Amoebiasis
Hippo signaling pathyway
Regulation of action cytoskeleton
TNF signaling pathway
Herpes simplex infection
HTLV-I infection
Ribosome biogenesis in eukaryotes
0
2
4
6
8
10
12
14
16
-Log10(q-value)

B

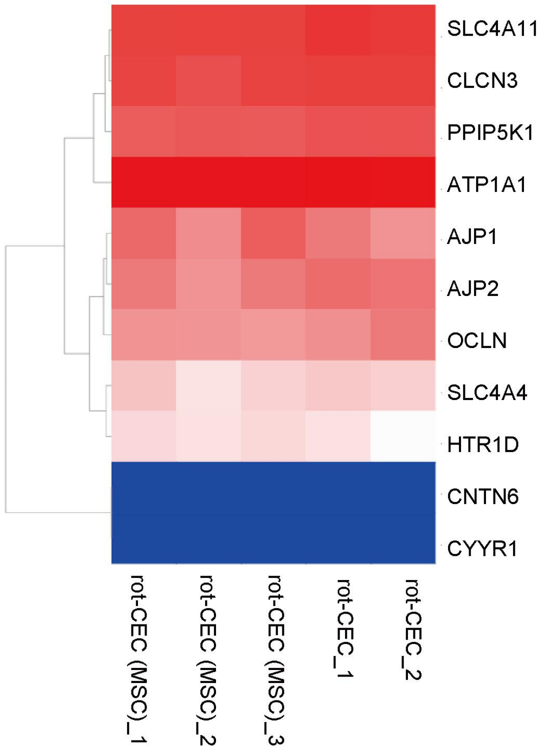
SLC4A11
CLCN3
PPIP5K1
ATP1A1
AJP1
AJP2
OCLN
SLC4A4
HTR1D
CNTN6
CYYR1
rot-CEC (MSC)_1
rot-CEC (MSC)_2
rot-CEC (MSC)_3
rot-CEC_1
rot-CEC_2

Figure S5

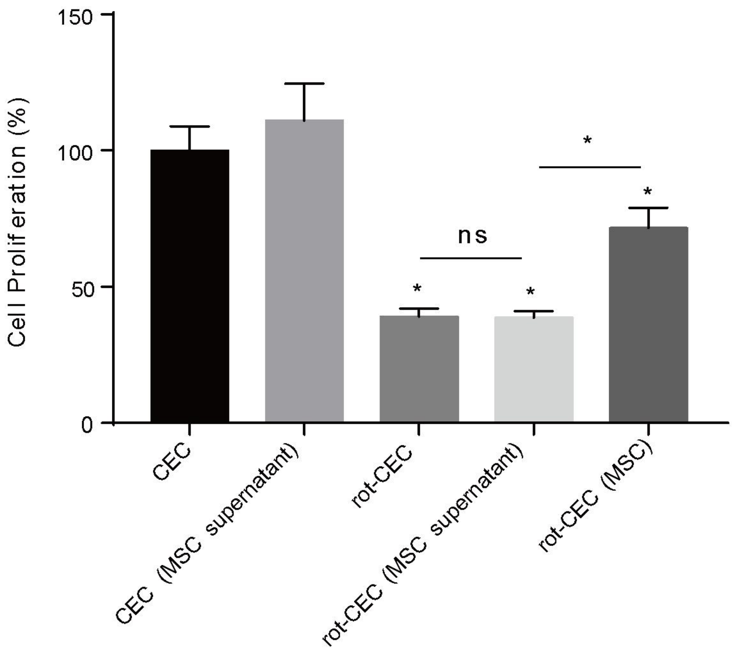

Figure S6

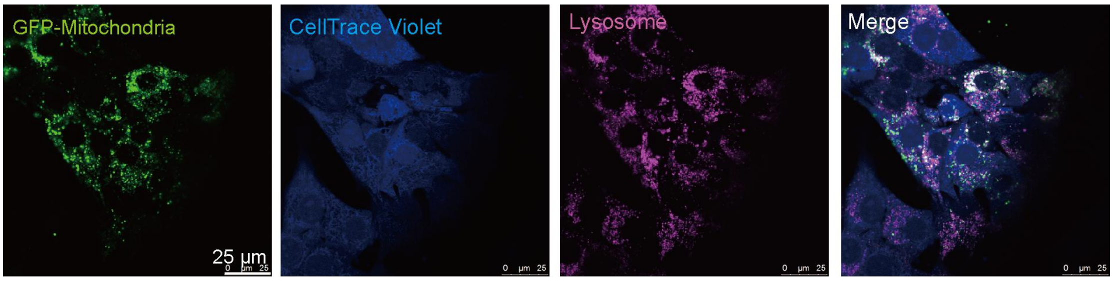

Supplement: Supplementary file 1 — Supplementary figures and tables. [file thnov10p7260s1.pdf]
